# Age-dependent gender differences in the diagnosis and treatment of osteoporosis during hospitalization in patients with fragility fractures

**DOI:** 10.1186/s12877-023-04430-y

**Published:** 2023-11-09

**Authors:** Qingmei Li, Jiancheng Yang, Qinghua Tang, Yan Feng, Mingming Pan, Maohong Che, Jie Shi, Yuhong Zeng

**Affiliations:** Department of Osteoporosis, Honghui Hospital, Xi’an Jiaotong University, No. 555 Youyi East Road, NanShaomen, Beilin District, Xi’an, 710054 China

**Keywords:** Fragility fracture, Osteoporosis, Bone mineral density, Bone metabolism biochemical markers, Anti-osteoporosis medications, Diagnosis, Treatment

## Abstract

**Background:**

There is a gender difference in the acceptance of osteoporosis diagnosis and treatment in patients after fragility fractures, but this difference is rarely assessed during hospitalization, and it is unclear whether these differences are age-dependent. This study aimed to evaluate the differences between male and female fragility fracture patients of different age groups who received the diagnosis and treatment of osteoporosis during hospitalization.

**Methods:**

31,265 fragility fracture patients aged ≥ 50 years from the Fragility Fracture Management Database in a high-volume orthopedic hospital from December 2019 to February 2023 were included in this study. We compared the differences in the rates of men and women with fragility fracture who received the measurement of bone mineral density (BMD) and bone metabolism biochemical markers (BMBMs) and treatment with anti-osteoporosis medications (AOMs), and follow-up to the internal medicine clinic within 3 months after discharge, across all age groups and across different age stages (50–59, 60–69, 70–79, and ≥ 80 years).

**Results:**

The detection rates of female patients receiving BMD and BMBMs during hospitalization were 31.88% and 5.30%, respectively, compared with 22.23% and 2.69% for men. The rate of receiving any AOMs treatment was 44.63% for women and 31.60% for men. The follow-up rate of returning to the internal medicine clinic within 3 months after discharge was 9.79% for women compared to 3.00% for men. There was a significant difference between males compared to females (*P* < 0.0001). Analysis of patients by different age group revealed that differences in the diagnosis and treatment of osteoporosis were found only in patients under 80 years of age, while gender differences in the return to the internal medicine clinic for follow-up after discharge were present in all age groups.

**Conclusions:**

Gender differences present in osteoporosis management in patients with fragility fracture during hospitalization, especially for patients under 80 years of age. This finding suggests that orthopedic surgeons neglect to manage osteoporosis in male patients with fragility fracture during hospitalization.

## Introduction

Osteoporotic fracture, the most serious complication of osteoporosis, is also known as fragility fracture. The number of people worldwide at risk for osteoporotic fractures aged 50 years or older was estimated to be 158 million in 2010 and will double by 2040 [1]. Fragility fracture induce pain and disability, leading to a significant reduction in quality of life, especially in the spine and hip, and can also reduce life expectancy, resulting in a significant social and medical burden [2]. High mortality associated with severe fragility fracture. Long-term bedridden patients with hip fragility fracture have a high mortality rate of 20–30% at 6 to 12 months and a permanent disability rate of more than 50% [3]. The risk of subsequent fractures in patients with fragility fracture was significantly increased. The incidence of secondary fracture was 7.58% in the first year and 11.58% in the first two years [4]. Therefore, it is crucial to initiate AOMs treatment as early as possible in patients with fragility fractures to prevent secondary fractures.

Despite various effective pharmacological interventions and well-established fracture prevention guidelines, most patients with fragility fracture do not receive BMD testing and AOMs treatment. This less frequent BMD testing and undertreatment is more common in men than in women. An evidence-based study showed BMD testing and anti-osteoporosis treatment rates for men after fracture are half of those for women [5]. A population-based retrospective cohort study showed that the detection rate of BMD and the use rate of AOMs within one year after fracture were 13.1% and 29.7% in women over 65 years of age, compared to only 4.6% and 9.9% in men [6]. Kiebzak et al [7] observed that 27% of male patients with hip fragility fracture received any AOMs treatment and 11% of men had bone densitometry at follow-up 1 to 5 years after hospital discharge, compared to 71% and 27% of women, respectively. However, few studies have evaluated gender differences in the diagnosis and treatment of osteoporosis in fragility fracture patients during hospitalization, and it is unclear whether these differences are age-dependent. This study retrospectively analyzed inpatient information on fragility fracture patients at a high-volume orthopedic hospital and compared the differences in the diagnosis and treatment of osteoporosis between female and male patients at different ages. Differences in the rate of female versus male fragility fracture patients returning to the internal medicine clinic for follow-up within 3 months after discharge were also analyzed.

## Methods

### Data source and study population

The present study protocol was reviewed and approved by the institutional review board of Honghui Hospital, Xi’an Jiaotong University (Reg. No. 202,305,003). Hong hui Hospital is a high volume orthopedic hospital located in northwest China. In order to better manage fragility fracture patients, our hospital established the fragility fracture management database in 2019, which has been synchronized with the hospital information system (HIS). The information on all hospitalized patients who may have experienced fragility fracture or have been diagnosed with osteoporosis will be updated in this database in a timely manner. From patients who were enrolled in the database between December 2019 and February 2023, 31,265 inpatients with fragility fracture were involved in this study based on the following inclusion and exclusion criteria. Inclusion criteria: (1) age ≥ 50 years; (2) no or only slight trauma; (3) all diagnosed as fragility fracture. Exclusion criteria: (1) age < 50 years; (2) serious violent trauma, such as falling from height, car accident, and high-energy impact injury, etc.; (3) fractures of skull, fingers and toes; (4) pathological fracture caused by primary or metastatic tumor.

As shown in Table 1, a total of 20,983 women, aged 70.88 ± 10.91 years, were included in this study, including 10,296 spine fractures, 6,751 hip fractures, 2,091 forearm fractures, 1,207 proximal humerus fractures, and 638 other fractures (including tibia, fibula, shaft of femur, patella, heel, ankle, clavicle, scapula, and ribs). A total of 10,282 males, aged 67.38 ± 12.13 years, were included, including 4,941 spinal fractures, 3,845 hip fractures, 825 forearm fractures, 425 proximal humerus fractures, and 246 other fractures.

**Table 1:** Patient characteristics

Characteristics	All patients	Female	Male
Total number, n (%)	31,265 (100)	20,983 (67.11)	10,282 (32.89)
**Basic information**			
Age (years), mean (SD)	69.73 (11.45)	70.88 (10.91)	67.38 (12.13)
Postmenopause, n (%)	18,905 (60.47)	18,905 (90.10)	N/A
Height (cm), mean (SD)	163.42 (7.61)	160.05 (6.18)	170.42 (5.11)
Weight (kg), mean (SD)	59.92 (11.37)	57.21 (9.97)	66.11 (11.94)
BMI (kg/m2), mean (SD)	22.25 (3.93)	22.15 (3.91)	22.47 (3.96)
**Previous history**			
Smoking history, n (%)	1871 (5.98)	89 (0.42)	1782 (17.33)
Drinking history, n (%)	955 (3.05)	46 (0.22)	909 (8.84)
Diabetes, n (%)	4646 (14.86)	3257 (15.52)	1389 (13.51)
Tumor history, n (%)	1078 (3.45)	693 (3.30)	385 (3.74)
COPD history, n (%)	344 (1.10)	150 (0.71)	194 (1.89)
**Fracture site**			
Spine, n (%)	15,237 (48.74)	10,296 (49.07)	4941 (48.05)
Hip, n (%)	10,596 (33.89)	6751 (32.17)	3845 (37.40)
Forearm, n (%)	2916 (9.33)	2091 (9.97)	825 (8.02)
Proximal humerus, n (%)	1632 (5.22)	1207 (5.75)	425 (4.13)
Other fractures, n (%)	884 (2.83)	638 (3.04)	246 (2.39)

*BMI* Body Mass Index, *COPD* chronic obstructive pulmonary disease

### BMD testing

BMD at the lumbar spine, femoral neck and total hip was measured by dual-energy X-ray absorptiometry (DXA) (Hologic Discovery-W or Hologic Horizon-A, Waltham, MA, USA). All DXA scanning were performed by the same certified radiologist.

### Serum biochemical assay

On the morning following an overnight fast, a blood sample is collected from the patient’s median cubital vein. Serum was obtained by centrifugation of blood samples. Serum calcium (Ca), phosphorus (P), and BMBMs, including type I procollagen N-terminal peptide (P1NP), type I collagen C-terminal peptide (CTX), 25-hydroxyvitamin D (25-OH-VD) and parathyroid hormone (PTH), were measured using a Roche Cobas 600 chemiluminescence apparatus.

### Anti-osteoporosis Medication

During hospitalization, one or more of Ca supplements, vitamin D (VD) supplements, active VD (alphacalcitol or calcitriol), calcitonin (salmon calcitonin or escitonin), bisphosphonates (alendronate sodium, risedronate sodium, ibandronate sodium, or zoledronic acid), denosumab, and traditional Chinese medicines (Xianling Gubao Capsule, Jintiange Capsule, Qianggu Capsule, or Gushukang Capsule) were used for anti-osteoporosis treatment.

### Statistical analysis

All data statistics were performed using GraphPad Prism 7 software. Measurement data of normal distribution were expressed as mean ± standard deviation, and the between-group differences in the counting data of each group were statistically analyzed by chi-square (*χ*^2^) test. A *P* value < 0.05 was considered significant.

## Results

### Bone mineral density and bone metabolism biochemical markers

As shown in Table 2, among 20,983 female patients, 6,690 (31.88%), 18,621 (88.74%) and 1,113 (5.30%) were detected for BMD, serum levels of Ca/P and BMBMs, respectively; among 10,282 male patients, 2,286 (22.23%), 8,899 (86.55%) and 277 (2.69%) were examined for BMD, serum levels of Ca/P and BMBMs, respectively. By comparison, it was found that the detection rates of BMD and serum levels of BMBMs were significantly lower in male patients with fragility fracture than in female patients (*P* < 0.0001).

**Table 2:** Assessments of bone mineral density and bone metabolism biochemical markers in patients with fragility fracture during hospitalization

Age, years	≥ 50			50–59			60–69			70–79			≥ 80		
Group	Female	Male	*P* value	Female	Male	*P* value	Female	Male	*P* value	Female	Male	*P* value	Female	Male	*P* value
Cases, n	20,983	10,282	N/A	3,839	3,495	N/A	5,977	2,644	N/A	5,623	1,963	N/A	5,544	2,180	N/A
BMD, n (%)	6,690 (31.88)	2,286 (22.23)	< 0.0001	1,372 (35.74)	716 (20.49)	< 0.0001	2,319 (38.80)	698 (26.40)	< 0.0001	1870 (33.26)	451 (22.98)	< 0.0001	1,129 (20.36)	421 (19.31)	0.2986
Ca/P, n (%)	18,621 (88.74)	8,899 (86.55)	< 0.0001	3,316 (86.38)	2,882 (82.46)	< 0.0001	5,270 (88.17)	2,312 (87.44)	0.3383	5,051 (89.83)	1,733 (88.28)	0.0554	4,984 (89.90)	1,972 (90.46)	0.4594
BMBMs, n (%)	1,113 (5.30)	277 (2.69)	< 0.0001	190 (4.95)	78 (2.23)	< 0.0001	397 (6.64)	58 (2.19)	< 0.0001	340 (6.05)	64 (3.26)	< 0.0001	186 (3.35)	77 (3.53)	0.6992

*BMD* bone mineral density, *Ca/P* calcium/phosphorus, *BMBMs* bone metabolism biochemical markers

To figure out whether these discrepancies between men and women is age-related, we analyzed the differences in BMD and BMBMs detection rates between men and women with fragility fracture at different ages. As shown in Table 2, between the ages of 50 and 79 years, the detection rates of BMD and the serum levels of BMBMs were significantly lower in men with fragility fracture than in women. However, in patients aged 80 years and older, the rate of BMD measurement and BMBMs testing was not significantly different compared with women. Gender differences in serum Ca/P levels were determined only in fragility fracture patients aged 50–59 years. Therefore, the proportion difference in detecting BMD and BMBMs between male and female patients with fragility fracture is related to age.

### The treatment with anti-osteoporosis medications

As shown in Tables 3, 11,094 of 20,983 male patients received any AOMs (including Ca and VD supplements), with a treatment rate of 52.87%; among them, the number of those who received Ca and/or VD supplements, active VD, calcitonin, bisphosphonates, denosumab, and traditional Chinese medicines were 3,259 (15.53%), 5,360 (25.54%), 6,276 (29.91%), 1,831 (8.73%), 152 (0.72%) and 240 (1.14%), respectively. Of the 10,282 male patients, 4,200 received any AOMs (40.85%); 1503 (14.62%), 1600 (15.56%), 2241 (21.80%), 474 (4.61%), 37 (0.36%) and 116 (1.13%) received Ca and/or VD supplements, active VD, calcitonin, bisphosphonates, denosumab, and traditional Chinese medicine, respectively. By comparison, it was found that male patients with fragility fracture received significantly lower rates of various types of AOMs than female patients (*P* < 0.0001), except for traditional Chinese medicine.

**Table 3:** Anti-osteoporosis drug treatment in patients with fragility fracture during hospitalization

Age, years	≥ 50			50–59			60–69			70–79			> 80		
Group	Female	Male	*P* value	Female	Male	*P* value	Female	Male	*P* value	Female	Male	*P* value	Female	Male	*P* value
Cases, n	20,983	10,282	N/A	3,839	3,495	N/A	5,977	2,644	N/A	5,623	1,963	N/A	5,544	2,180	N/A
Any anti-OP drug, n (%)	11,094 (52.87)	4,200 (40.85)	< 0.0001	1,732 (45.12)	1,099 (31.44)	< 0.0001	3,354 (56.12)	1,091 (41.26)	< 0.0001	3,222 (57.30)	940 (47.89)	< 0.0001	2,786 (50.25)	1,071 (49.13)	0.3738
Ca and/or ordinary VD, n (%)	3,259 (15.53)	1,503 (14.62)	0.0346	609 (15.86)	506 (14.48)	0.0988	935 (15.64)	381 (14.41)	0.1420	903 (16.06)	281 (14.31)	0.0668	797 (14.38)	305 (14.00)	0.6632
Active VD, n (%)	5,360 (25.54)	1,600 (15.56)	< 0.0001	779 (20.29)	327 (9.36)	< 0.0001	1,761 (29.46)	419 (15.85)	< 0.0001	1,636 (29.09)	397 (20.22)	< 0.0001	1,184 (21.36)	457 (20.96)	0.7038
Calcitonin, n (%)	6,276 (29.91)	2,241 (21.80)	< 0.0001	873 (22.74)	432 (12.36)	< 0.0001	1,866 (31.22)	581 (21.97)	< 0.0001	1,856 (33.01)	548 (27.92)	0.0007	1,681 (30.32)	641 (29.40)	0.4287
Bisphosphonates, n (%)	1,831 (8.73)	474 (4.61)	< 0.0001	273 (7.11)	69 (1.97)	< 0.0001	657 (10.99)	145 (5.48)	< 0.0001	521 (9.27)	135 (6.88)	0.0012	380 (6.85)	119 (5.46)	0.0247
Denosumab, n (%)	152 (0.72)	37 (0.36)	< 0.0001	16 (0.42)	11 (0.31)	0.4711	58 (0.97)	10 (0.38)	0.0042	48 (0.85)	8 (0.41)	0.0468	30 (0.54)	8 (0.37)	0.3248
Chinese medicine, n (%)	116 (1.13)	240 (1.14)	0.9028	78 (2.03)	74 (2.12)	0.7973	65 (1.09)	23 (0.87)	0.3540	47 (0.84)	11 (0.56)	0.2277	50 (0.90)	8 (0.37)	0.0142

*OP* osteoporosis, *Ca* calcium, *VD* vitamin D

To determine whether these differences between men and women is age-related, we analyzed the discrepancies in the utilization rate of AOMs between men and women with fragility fracture at different ages. As shown in Table 3, between the ages of 50 and 79 years, male patients with fragility fracture received any AOMs, active VD, calcitonin, and bisphosphonates at a significantly lower rate than women, whereas there was no significant difference between men and women aged 80 years or older. There was no significant difference between men and women receiving Ca and/or VD supplements at any age. Patients prescribed with denosumab for anti-osteoporosis showed only modest gender disparities between the ages of 60 and 79. There is only a mild difference between men and women aged 80 or older who receive traditional Chinese medicine for anti-osteoporosis treatment. Thus, the difference between male and female patients with fragility fracture receiving various types of AOMs during hospitalization is age-dependent.

### Follow-up rate of coming back to the internal medicine clinic after hospital discharge

As shown in Table 4, the rate of follow-up to the internal medicine clinic within 3 months after discharge for male patients with fragility fracture (3.00%) was significantly lower than that of female patients (9.79%). By analyzing the follow-up rate of patients of different age groups, it was found that the follow-up rate of male patients with fragility fracture at any age group to the internal medicine clinic after discharge was significantly lower than that of female patients (*P* < 0.0001).

**Table 4:** The follow-up rate of patients with fragility fracture to the internal medicine clinic after discharge

Age, years	Group	Cases, n	Follow-up rate, n (%)
≥ 50	Female	20,983	2,054 (9.79)
	Male	10,282	308 (3.00)
	*P* value	N/A	< 0.0001
50–59	Female	3,839	356 (9.27)
	Male	3,495	76 (2.17)
	*P* value	N/A	< 0.0001
60–69	Female	5,977	723 (12.10)
	Male	2,644	85 (3.21)
	*P* value	N/A	< 0.0001
70–79	Female	5,623	635 (11.30)
	Male	1,963	68 (3.46)
	*P* value	N/A	< 0.0001
≥ 80	Female	5,544	350 (6.31)
	Male	2,180	79 (3.62)
	*P* value	N/A	< 0.0001

## Disscussion

BMD is the “gold standard” for diagnosing osteoporosis and is the primary indicator for assessing fracture risk. Evaluation of BMD in fragility fracture patients can significantly increase the rate of anti-osteoporosis treatment [8]. However, most patients with fragility fractures do not have a BMD testing after the fracture, and this is more severe in men [5, 9, 10]. For example, a retrospective cohort study based on 25,852 individuals showed that the rate of women aged > 65 years with fragility fracture receiving BMD testing within one year after fracture was 13.1%, compared to 4.6% for men [6]. Consistent with these findings, our results showed that the rate of BMD detection during hospitalization was 22.23% in men with fragility fracture, which was significantly lower than 31.88% in women. It is not clear why male patients usually receive fewer BMD exams after fragility fracture than women. Fewer guidelines and consensus regarding men with osteoporosis or fragility fracture, the lack of social emphasis on screening for osteoporosis in men, and the lack of awareness among physicians and patients may explain this phenomenon to some extent [11, 12]. In conclusion, male patients with fragility fracture frequently have inadequate BMD screening compared to women.

BMBMs include those enzymes and hormones secreted by endocrine glands or bone tissue that can regulate bone metabolic processes, as well as collagen metabolites or non-collagen proteins derived from the bone matrix [13]. By measuring BMBMs levels in blood and urine, it can be used to evaluate bone metabolic status, differential diagnosis of metabolic bone disease, diagnostic typing of osteoporosis, assessment of osteoporosis treatment effect and prediction of fracture risk [14, 15]. To our knowledge, no studies have been reported on BMBMs in fragility fracture patients during hospitalization. Our data demonstrated that male fragility fracture patients have a lower detection rate of BMBMs than female patients, including P1NP, CTX, 25-OH-VD, and PTH, other than serum Ca/P during hospitalization. It is not difficult to find that the detection rate of BMBMs is significantly lower than BMD testing rate. The possible reason is that BMBMs are not the gold standard for the diagnosis of osteoporosis, leading orthopedic surgeons to neglect the testing of these indicators. The results of this study showed the prevalence of testing serum Ca/P levels in fragility fracture patients by orthopedists. The main reason is that serum Ca/P levels not only reflect the skeletal metabolism, but also are key indicators to evaluate the electrolyte balance of the body. The assessment of electrolytes can help orthopedists to quickly and accurately develop the patient’s surgical treatment program, therefore, the assessment of the body’s electrolytes is a must prior to surgery.

The treatment of AOMs after fragility fracture is important to prevent secondary fractures and reduce mortality [16, 17]. However, numerous studies have reported lower use of anti-osteoporosis drugs after fragility fracture, and significantly lower in men than in women [18–24]. For example, Vanasse et al. [6] analyzed data on fragility fracture patients older than 65 years in Quebec, Canada, in 1999 and 2000, and found that the rate of female patients receiving AOMs within one year after fracture was 29.7%, compared to 9.9% for men. Recently, a retrospective cohort study of fragility fracture patients from 37 hospitals in Fujian Province, China, showed that 22.1% of women and 9.5% of men aged 50 years or older received anti-osteoporosis treatment within 1 year after fragility fracture between 2010 and 2016, with 5.3% of women and 1.5% of men using bisphosphonates [25]. In conclusion, all of these findings suggest that men with fragility fracture tend to use AOMs after fracture at a lower rate than women, which is consistent with the data from present study. We found that male fragility fracture patients received 31.60% of any AOMs during hospitalization compared to 44.63% of women, and men were significantly less possibility than women to use active VD, calcitonin, bisphosphonates or denosumab.

It is well known that the occurrence and outcome of both fragility fracture and osteoporosis are closely related to age [26, 27]. Age is also usually an important consideration for physicians in the diagnosis and treatment of patients. Recently, Hoit et al [28] analyzed hip fracture patients from the American College of Surgeons National Surgical Quality Improvement Program registry between 2016 and 2018 and found that male patients in their 50s, 60s, and 70s were less likely to be taking AOMs compared to women in the same age group. However, for people aged 80 years or older, there was no difference in treatment between men and women. Consistently, the data from this study reveal a similar phenomenon in that gender differences in fragility fracture patients treated with AOMs were found only in patients younger than 80 years. In addition, this study revealed a similar situation in the detection of BMD and BMBMs in fragility fracture patients. Our results showed that male patients aged 50–59, 60–69, and 70–79 years received markedly lower rates of BMD and BMBMs testing than women in the same age groups. For patients aged 80 years and older, there were no significant differences between the sexes for BMD and BMBMs exams. To our knowledge, this study is the first to assess the effect of age on sex differences in the diagnosis of osteoporosis in fragility fracture patients.

This study showed that for the treatment of osteoporosis in fragility fracture patients during hospitalization, the use of calcium and common VD, active VD and calcitonin is much higher than the use of other drugs. Calcium and common VD are the basic supplements for the treatment of osteoporosis and are often used together with other AOMs [28]. Calcitonin and active VD are widely used probably due to their good safety profile, low contraindications to their use and low cost. In addition, another reason why calcitonin is heavily used is its ability to significantly relieve bone pain caused by osteoporosis or fractures [29]. Bisphosphonates, denosumab, and teriparatide have been shown to have a clear reduction in the risk of osteoporotic fractures and are the preferred recommended anti-osteoporosis drugs in various national guidelines related to osteoporosis and fragility fractures [30, 31]. Our study found very low use of bisphosphonates and denosumab, and no use of teriparatide was even observed during hospitalization. A reasonable explanation is that these drugs often have multiple contraindications to administration and transient adverse effects following administration, and require long-term management after use, leading to unfamiliarity and reluctance among orthopaedic surgeons to use these drugs. In addition, Chinese health insurance policies and our hospital policies for different AOMs during hospitalization have been associated with low utilization of these first-line AOMs. Therefore, it is very important for patients to be followed up at the internal medicine clinic after discharge from the hospital.

There are many types of osteoporosis, and different types of osteoporosis have different treatment methods, especially secondary osteoporosis, for which special treatment plans should be developed to address the cause of the induced osteoporosis. In addition, the variety of anti-osteoporosis drugs, the many contraindications to their use and the various side effects that can occur with long-term use make it necessary to standardize and implement long-term management of AOMs treatment [32]. Therefore, the differential diagnosis of osteoporosis requires extensive basic medical knowledge and medication experience, which is often lacking in orthopedic surgeons. For this reason, the International Osteoporosis Foundation recommends the fracture liaison service (FLS) model, which connects orthopedists and endocrinologists through a liaison or coordinator for the professional treatment, care and follow-up of patients with fragility fractures [33]. However, FLS has not been widely implemented in China. Therefore, it remains a relatively popular phenomenon in China, at least in our hospital, for fragility fracture patients to return voluntarily to the osteoporosis specialist or endocrine clinic for follow-up after discharge. In this study, we found a significant gender difference in the follow-up rate of fragility fracture patients returning to the internal medicine clinic within 3 months of discharge, with 3% of men and 9.79% of women. And this difference persists across age groups. The lower follow-up rate in male patients may be related to the psychological and personality characteristics of men. Men generally have high self-esteem and are reluctant to admit their weakness. As a result, they cannot accept or acknowledge that their bones are weakening, leading to a lack of attention and poor adherence to the diagnosis and treatment of osteoporosis.

## Conclusions

Although all data in this study are from the same hospital and it is uncertain whether they are representative of other hospitals or regions, gender differences in the diagnosis and treatment of osteoporosis in patients with fragility fractures are, in any case, a common phenomenon, especially for patients under 80 years of age. The clinical implications of these findings are that strategies to increase the diagnosis of osteoporosis and the prescription of AOMs should be more frequent in elderly patients hospitalized for fragility fracture, especially in men. On the other hand, this gender difference indicates that society, orthopaedic surgeons and patients themselves are far from paying enough attention to osteoporosis in men, and therefore increased social awareness about osteoporosis in men, training for orthopedists and health education for male patients may be one way to reduce this discrepancy.

## Data Availability

The datasets generated and/or analysed during the current study are not publicly available due to the limitations of our hospital’s information security and confidentiality system, but data are available from the corresponding author (xahhzyh@163.com) with the appropriate approval from the Institutional Review Board of Honghui Hospital, Xi’an Jiaotong University.

## Abbreviations

BMD: bone mineral density
BMBMs: bone metabolism biochemical markers
AOMs: anti-osteoporosis medications
DXA: dual-energy X-ray absorptiometry
P1NP: type I procollagen N-terminal peptide
CTX: type I collagen C-terminal peptide
25-OH-VD: 25-hydroxyvitamin D
PTH: parathyroid hormone
FLS: fracture liaison service
